# Syndromic epidermolysis bullosa simplex subtype due to mutations in the *KLHL24* gene: series of case reports in Russian families

**DOI:** 10.3389/fmed.2024.1418239

**Published:** 2024-07-29

**Authors:** Yulia Y. Kotalevskaya, Vadim A. Stepanov

**Affiliations:** ^1^Medical Genetics Center, Vladimirsky Moscow Regional Research and Clinical Institute, Moscow, Russia; ^2^Research Institute of Medical Genetics, Tomsk National Research Medical Center of the Russian Academy of Sciences, Tomsk, Russia

**Keywords:** epidermolysis bullosa simplex, syndromic epidermolysis bullosa, rare phenotype, genodermatosis, *KLHL24* gene, genotype–phenotype correlations

## Abstract

**Objective:**

Epidermolysis bullosa simplex (EBS) is a common, well-characterized type of epidermolysis bullosa. However, some rare syndromic EBS phenotypes are not well described. The accumulation of clinical descriptions of patients with syndromic subtypes of EBS is important for understanding the natural history of the disease and assessing genotype–phenotype correlations.

**Case summary:**

We present a series of case reports of the syndromic subtype of EBS associated with mutations in the *KLHL24* gene in seven patients from four unrelated families. The clinical features of this rare phenotype in children and adult patients are described in detail. In two families, we revealed pathogenic variant c.1A > G (p.Met1?) in the *KLHL24* gene. The third family had c.3G > A (p.Met1?) mutation, and the fourth family had a novel *de novo* variant c.23del (p.Arg8AsnfsTer2).

**Conclusion:**

The description of the clinical manifestations of the disease in two generations of EBS families with different genetic variants allows the assessment and prediction of the natural course and severity of the disease in these families, the risk of complications, and the planning of the amount of medical care necessary.

## Introduction

Epidermolysis bullosa simplex (EBS) is one of the four main and most genetically heterogeneous types of epidermolysis bullosa (EB) and is caused by mutations in seven different genes (1). The most common subtypes result from mutations in the *KRT5* and *KRT14* genes, which are expressed in the basal layer of the epidermis and encode keratin intermediate filaments. In addition, mutations in the *PLEC*, *DST*, *EXPH5*, *KLHL24,* and *CD151* genes explain rare syndromic EBS subtypes (1, 2).

The main subtypes of EBS are inherited as autosomal dominant, *de novo* mutations have also been described (3).

Mutations in the *KLHL24* gene, which encodes for Kelch-like protein 24, have been detected by whole exome sequencing (WES) in patients with skin fragility and progressive nail thickening in whom mutations in other genes associated with EB have been excluded (4, 5). *KLHL24* is part of a family of over 40 genes with a Kelch-like motif that is part of a ubiquitin ligase complex of proteins with variable tissue expression patterns. These proteins are involved in the ubiquitination and proteasomal degradation of various substrates, including epidermal keratins (6).

Clinical manifestations of generalized intermediate EBS with or without cardiomyopathy (OMIM#617294) associated with mutations in the *KLHL24* gene include areas of skin aplasia on the hands and legs and heal with the formation of hypopigmentation, atrophic and stellate scars, and follicular atrophoderma. In childhood, generalized blistering, nail fragility, and progressive thickening are seen. In adults, there is mild skin fragility, hypopigmentation, thickening of the toenails, and diffuse hair loss. Young adult patients are at risk of developing dilated cardiomyopathy (3, 6).

To date, the pathogenetic mechanisms underlying this syndromic EBS phenotype have not been fully characterized. In some patients, the development of dilated cardiomyopathy at a young age has been described, resulting from KLHL24-mediated degradation of desmin in the intermediate filaments of cardiomyocytes (7).

Establishing a diagnosis of the syndromic EBS subtype associated with mutations in the *KLHL24* gene at birth or at an early age is extremely difficult, even for an experienced clinician. Information on the spectrum of clinical manifestations and the natural history of this subtype of EBS is accumulating, and molecular genetic research is playing a crucial role in clarifying the diagnosis. However, in the absence of molecular diagnostics, long-term natural history observation will be required to establish this subtype.

This report provides the first description of seven clinical cases from three unrelated Russian families in which the WES played a key role in clarifying the diagnosis.

## Cases presentation

### Case 1

Family 1 with 3 patients: a mother born in 1989 and 2 children—a girl born in 2009 and a boy born in 2015.

**Figure 1:**
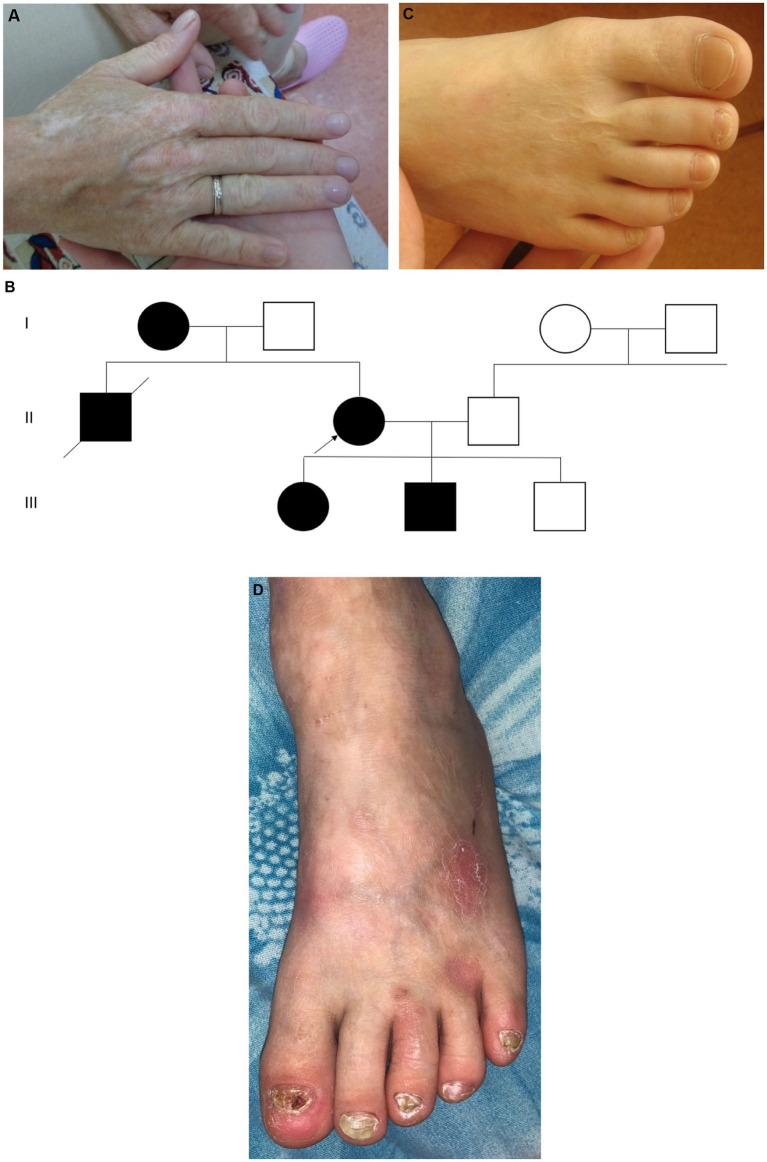
Case 1: **(A)** – mother – atrophic scars and hypopigmentation; **(B)** – pedigree of family 1; **(C)** – daughter – specific stellate scars on the dorsum of the foot; **(D)** – son – toenail dystrophy, hypopigmentation. Photo shared by the charitable foundation “BELA. Butterfly Children”. Reprinted with permission from Charitable fund “BELA. Butterfly Children”.

The mother was born from the 4th pregnancy, 2 births, birth weight was 3,000 g. At birth, she had skin fragility and areas of aplasia cutis on her hands and legs. Congenital EB was diagnosed at the maternity hospital. Throughout her life there has been a positive dynamic of the skin process, the blisters rarely occurred around preschool age or only with mechanical trauma, and the atrophic scars developed. There were many areas of hypopigmentation on the limbs (Figure 1A), several areas of follicular atrophoderma on the hands, and in the neck area. The toenails were thickened, but the fingernails were normal. She has diffused alopecia and looks older than her age. She also has no history of heart problems in her life.

EB occurs in her family in three generations, a total of 5 patients are known: 1. the proband’s mother, died at the age of 46, cause unknown; 2. the proband’s brother, died at the age of 27, an accident, both had clinical symptoms consistent with the proband’s phenotype; 3. the proband; 4, 5. the proband’s children—daughter and son (Figure 1B).

The proband’s daughter was born in 2009 from the first pregnancy at 38–39 weeks of gestation with a birth weight of 3,320 g. There was aplasia cutis on the feet, thighs, hands, and elbows; in the neonatal period, the diagnosis was epidermolysis bullosa, dystrophic type. Like her mother, the daughter under the age of 5 years often developed blisters with fairly rapid epithelialization. After the age of 5 years, the blisters were extremely rare, but she had hypopigmentation on the limbs, atrophic scars on the hands, giving the skin an “aged” appearance, and stellate scars on the feet (Figure 1C). At the age of 8 years, the number of hypopigmented areas increased, and toenail dystrophy and diffuse alopecia appeared. At the age of 12 years, the clinical picture remained stable.

The boy was born in 2015 from a second pregnancy at 39 weeks of gestation with a birth weight of 2,760 g. He had aplasia cutis on the anterior lower leg and hand surfaces. Epidermolysis bullosa of dystrophic type was also diagnosed. The dynamics of the skin process: up to 3 years, the blisters developed on the skin of the body and legs, and there was a long healing period of erosions. He had atrophic scars, hypopigmented skin, and areas of follicular atrophoderma on the legs. Nail dystrophy was noted, especially in the toenails (Figure 1D). At 5 years of age, typical stellate scars on the palms, as well as numerous areas of hypopigmentation on the limbs and back, were observed. The skin of the limbs has an “aged” appearance and the hair is sparse.

WES was performed from the boy’s DNA sample, and the heterozygous pathogenic variant c.1A > G (p.Met1?) in the *KLHL24* gene was revealed. This heterozygous pathogenic variant was confirmed using Sanger sequencing in the proband, her daughter, and her son. All members of the family have been diagnosed with EBS associated with mutations in the *KLHL24* gene.

### Case 2

**Figure 2:**
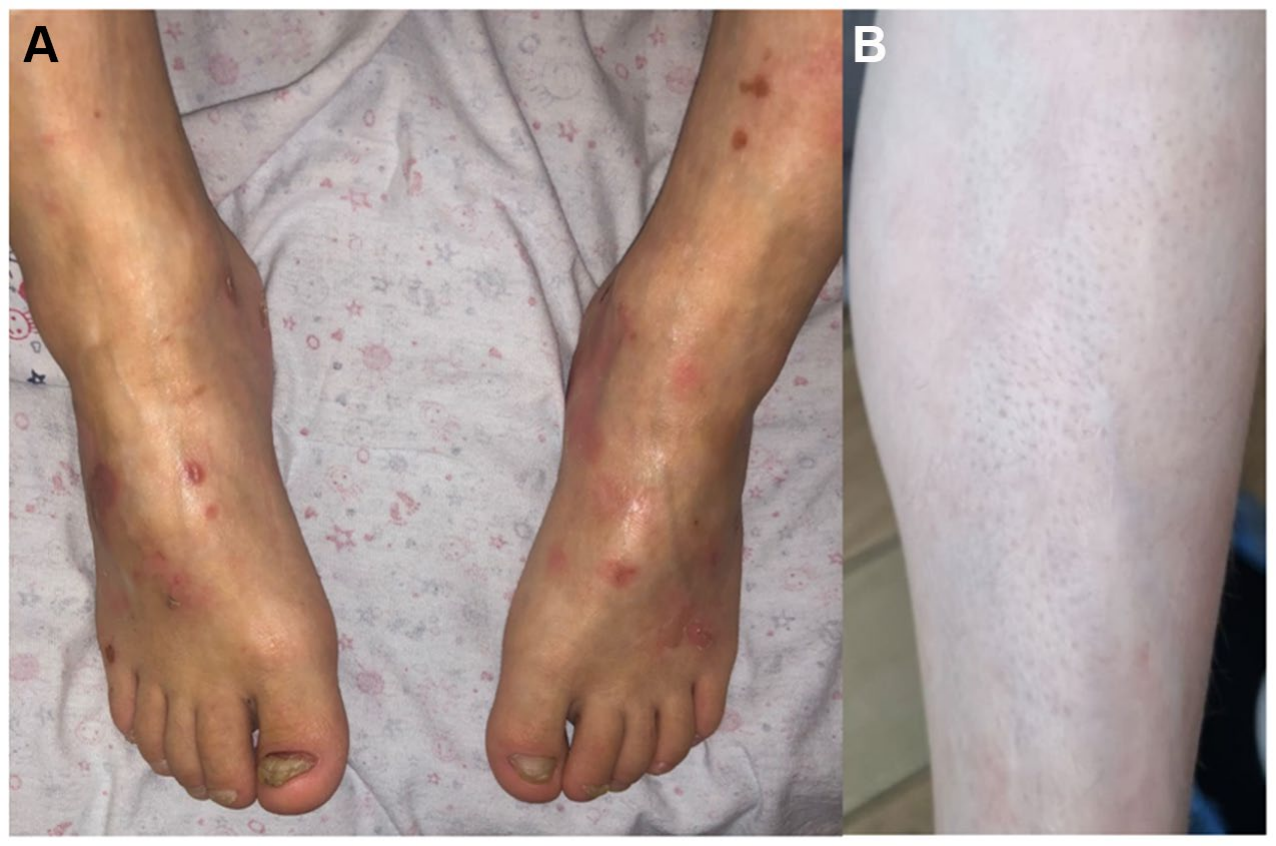
Case 2: **(A)** toenail dystrophy and small erosions. Photograph shared by the charitable foundation “BELA. Butterfly Children”; Reprinted with permission from Charitable fund “BELA. Butterfly Children”. **(B)** follicular atrophoderma.

Family 2, single patient—girl, born in 2015 to healthy parents, from the first pregnancy. She had areas of aplasia cutis on the wrist, dorsum of the palm, and dorsum of the foot, ankle, and knee joints. She was diagnosed with EB, probably dystrophic type. The dynamics of the skin process: after 1.5 months, blisters appeared in places of friction, under bandages, and on the oral mucosa. At the age of 6 months, she had hypopigmentation, atrophic scars, follicular atrophoderma on the legs, and milia. At the age of 2 years, there were isolated blisters and erosions on the body and oral mucosa. There were typical stellate scars on the dorsum of the hands and toenail dystrophy developed (Figure 2).

The heterozygous pathogenic variant c.1A > G (p.Met1?) in the *KLHL24* gene was detected by WES and confirmed as *de novo* in the proband.

### Case 3

**Figure 3:**
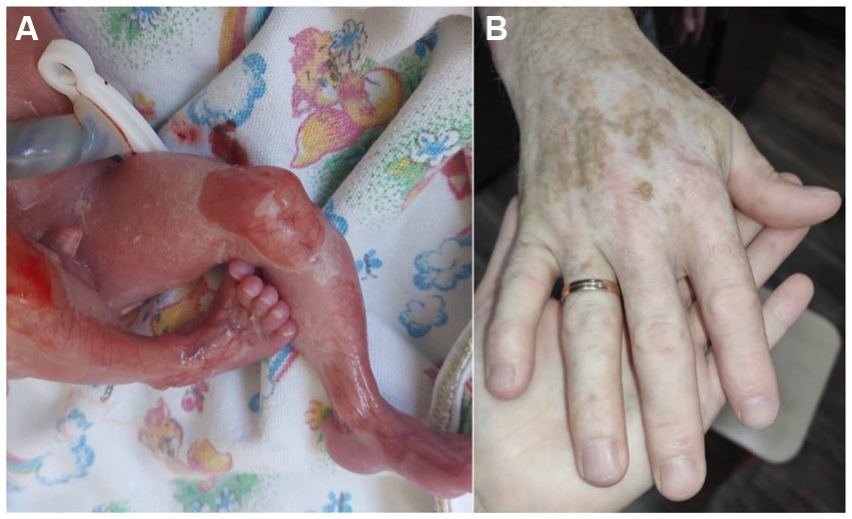
Case 3: **(A)** boy—low weight, areas of skin aplasia; **(B)** father—hypopigmentation.

Family 3 includes 2 patients—the son born in 2021 and the father born in 1992. The proband is a boy from the first pregnancy, which occurred against the background of gestosis. It was the first birth, delivered by an unexpected cesarean section in the 27th week of gestation. He had aplasia cutis on the legs, dorsum of the foot, and hands and a blister in the armpit (Figure 3A). During the course of treatment in the intensive care unit, large blisters and erosions developed due to the skin trauma associated with the treatment measures. The child was discharged home at the age of 4.5 months, at which time the areas of aplasia cutis were epithelialized, with rare blisters on the oral mucosa. He also had atrophic scars, milia, and areas of hypopigmentation. Up to the age of 6 months, he had areas of follicular atrophoderma on the legs, and his nails were normal.

The father of the proband was born in 1992 from the first pregnancy with a birth weight of 3,000 g. He also had skin aplasia on the limbs, erosions, and blisters and was diagnosed with a dystrophic type of EB. The blisters continued until he was 3 years old. At present, he has atrophic scars on the dorsum of the hands and legs, with more marked areas of hypopigmentation on the hands (Figure 3B). He also has sparse hair and normal nails. The patient had no cardiac problems and considered himself to be healthy.

We identified heterozygous pathogenic variant c.3G > A (p.Met1?) in the *KLHL24* using WES and then confirmed this variant using Sanger sequencing in the proband and his father.

### Case 4

**Figure 4:**
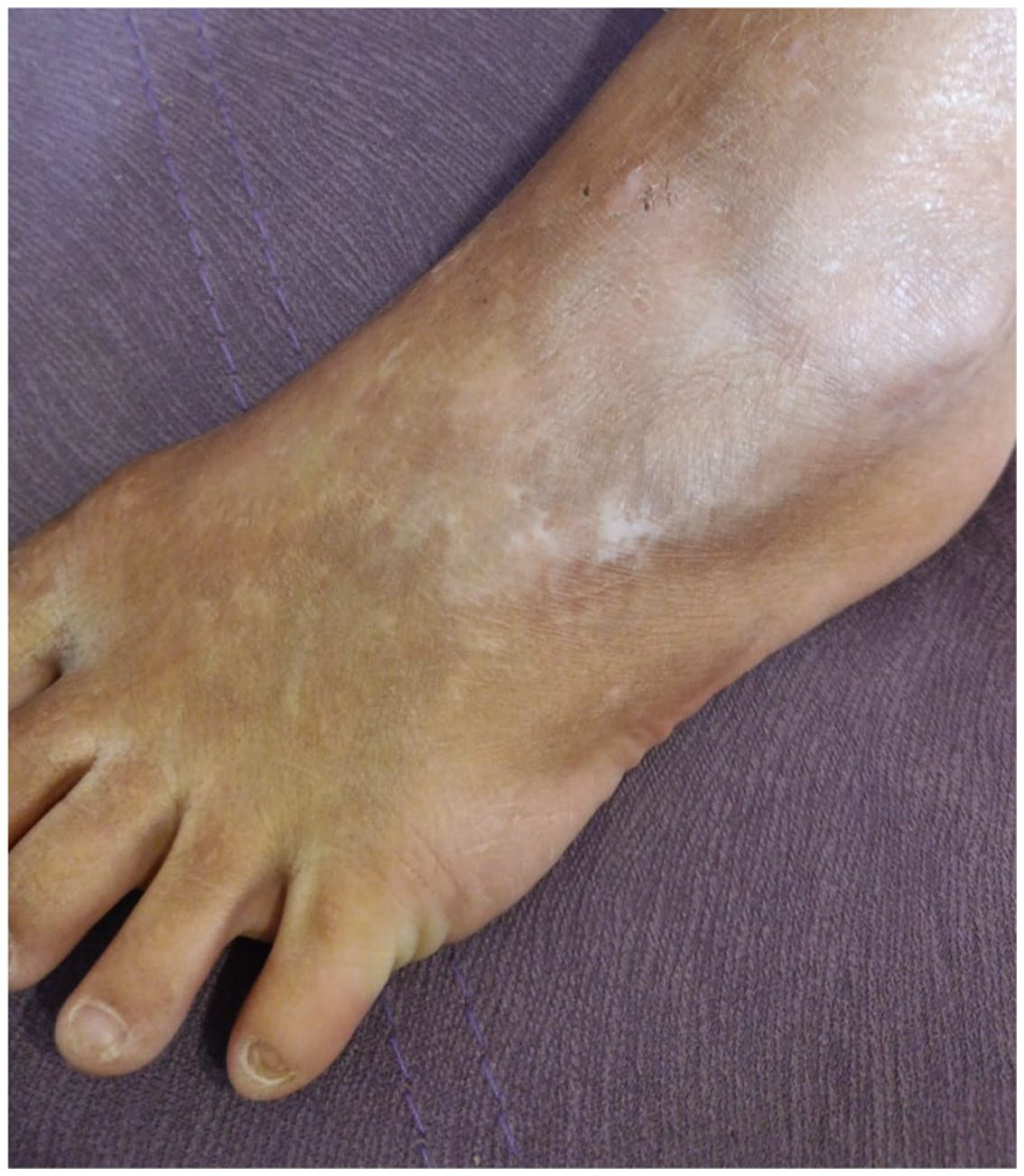
Case 4: (boy)—hypopigmentation, toenail dystrophy.

Family 4, 1 patient—a boy, born in 2011 to healthy parents from first pregnancy with a birth weight of 3,650 g. There were extensive areas of aplasia cutis on the abdomen, dorsum of the hands, and dorsum of the feet, ankles, and knees. Rapid epithelialization of the erosions was noted. Before the age of 1 year, blisters appeared at the places of friction, there were periodic lesions of the mucous membrane of the mouth and dystrophy of the nails of the little toes. In place of the erosions on the backs of the hands, typical star-shaped scars were formed (Figure 4). There is scar tissue on the abdomen at the site of aplasia and areas of hypopigmentation on the back and legs. Hair is normal. No cardiac manifestations were noted.

WES was performed on the proband and revealed a novel genetic variant c.23del (p.Arg8AsnfsTer2) in exon 4 of the *KLHL24* gene, which was confirmed as *de novo* by Sanger sequencing.

## Discussion and conclusion

The reviewed case series is the first description of patients with EBS associated with mutations in the *KLHL24* gene in Russia. This phenotype is extremely rare in patients with EB and represents a complex clinical challenge. The clinical manifestations of the disease described in our patients clearly correlate with clinical manifestations in patients from other populations (5).

Thus, in 2016, Lin et al. first described this phenotype in five unrelated EB patients, demonstrating the association of EB with *KLHL24* gene mutations (4). Immunofluorescent (IF) staining showed cleavage at the level of basal keratinocytes above the hemidesmosomes, close to the basement membrane, supporting the diagnosis of EBS. Has and Fischer then demonstrated an abnormal distribution of desmoplakin in the basal epidermal layer on IF (3). In addition, in 2016, He et al. described 14 additional patients from unrelated families with EB associated with mutations in the *KLHL24* gene from Germany, Switzerland, Finland, Qatar, and Italy (5). Transmission electron microscopy showed basal keratinocyte disruption and cytolysis. All described patients had heterozygous pathogenic variants in the translation start codon of the *KLHL24* gene, c.1A > G, c.1A > T, c.2 T > C, c.3G > T, c.3G > A, with a high frequency of these variants occurring *de novo* (3). In our study, we only did genetic testing, and the Russian patients had two known genetic variants: c.1A > G (p.Met1?), c.3G > A (p.Met1?) and a novel variant: c.23del (p.Arg8AsnfsTer2) in the *KLHL24* gene, two of which were *de novo*. The c.23del (p.Arg8AsnfsTer2) variant leads to frameshift mutations and is considered to be likely pathogenic.

Functional studies have shown that the presence of a pathogenic genetic variant in the *KLHL24* gene leads to the expression of a truncated protein. The pathogenetic mechanisms underlying this phenotype are still under investigation. Initially, it was found that mutations in the *KLHL24* gene indirectly affect the degradation of keratin 14 protein, leading to clinical manifestations (5). However, a more detailed study of the pathobiology of EBS associated with mutations in the *KLHL24* gene suggests that other mechanisms are important in the manifestation of the phenotype. Thus, in a study published in 2022, using a model of fetal keratinocytes, it was shown that mutations in the *KLHL24* gene lead to decreased levels of embryonic keratins 7, 8, 17, and 18 as a result of proteasomal degradation in normal fetal keratinocytes compared to control cells expressing the wild-type form of the *KLHL24* gene (7).

The main clinical manifestations of the disease are characterized by a certain staging and have different manifestations at different ages. Thus, according to the literature, all patients are born with areas of aplasia cutis on the limbs, less often on other parts of the body, and subsequently with the formation of atrophic scars in the areas of aplasia, often with a stellate scar pattern (3, 5, 8). These symptoms correlate with the clinical manifestations in Russian patients. In infancy, patients with this subtype develop areas of hypopigmentation and blistering at friction sites, with the incidence of blistering decreasing with age. Another pathognomonic sign of this subtype of EBS is the development of follicular atrophoderma, which is also seen in all Russian patients, appears in early childhood, and persists into adulthood. Other characteristic symptoms include thickening of the toenails and diffuse hair loss with age. We note that thickening of the toenails was not observed in only one of our adult patients. Perhaps the absence of this symptom is related to this genotype, which is different from other patients who have had changes in the toenails since childhood. In addition, dilated cardiomyopathy at a young age has been described in the literature in a number of patients. We did not find any cardiac pathology in our patients of different ages. In addition, in families with the c.1A > G (p.Met1?) genetic variant, a stable age-dependent phenotype was observed with a moderate course in children and adult patients, but in the third family with the c.3G > A (p.Met1?) genetic variant, the course of the disease was milder in the adult. In case 4, the patient had typical clinical EB symptoms only in childhood. After the age of 4 years, he had only skin healing results.

The therapeutic strategies for the treatment of EB are diverse and include complementary or synergistic combinations to target different symptoms such as fibrosis, pruritus, and skin cancer, including strategies to control systemic inflammation and others. Only our pediatric patients needed treatment, which included prevention of trauma and blistering, using atraumatic dressings and healing agents. Adult patients did not require treatment.

The monitoring of the dynamics of the clinical symptoms in our patients indicates a good prognosis for the skin process in this subtype of EBS and for the state of health in general. This knowledge reflects the importance of differential diagnosis, especially at an early age, because the accuracy of the diagnosis greatly influences the prognosis of the disease, which is important for the psychological state of the family and for determining the amount of medical care.

## Data availability statement

The original contributions presented in the study are included in the article/supplementary material, further inquiries can be directed to the corresponding author.

## Ethics statement

The studies involving humans were approved by the Research Institute of Medical Genetics, Tomsk National Research Medical Center of the Russian Academy of Sciences. The studies were conducted in accordance with the local legislation and institutional requirements. Written informed consent for participation in this study was provided by the participants’ legal guardians/next of kin. Written informed consent was obtained from the individual(s), and minor(s)’ legal guardian/next of kin, for the publication of any potentially identifiable images or data included in this article. Written informed consent was obtained from the participant/patient(s) for the publication of this case report.

## Author contributions

YK: Conceptualization, Data curation, Supervision, Writing – original draft, Writing – review & editing. VS: Supervision, Writing – review & editing.
